# The Role of Positron Emission Tomography for the Management of Sinonasal Malignancies: A Systematic Review

**DOI:** 10.1177/19458924231177854

**Published:** 2023-05-25

**Authors:** David El-Adem, Nathan Yang, David A. Gudis

**Affiliations:** 1 12367 Faculty of Medicine, McGill University, Montreal, Canada; 2 Department of Otolaryngology-Head & Neck Surgery, McGill University, Montreal, Canada; 3 Department of Otolaryngology-Head & Neck Surgery, 5798 Columbia University, New York City, United States

**Keywords:** positron emission tomography, sinonasal malignancies, skull base, cancer, sinus, imaging modality, diagnostic, staging, prognosis, surveillance

## Abstract

**Background:**

Positron emission tomography (PET) scan is a valuable imaging modality widely used in the management of cancers. Its usage is well defined for most head and neck malignancies. However, there is a lack of consensus regarding the utility of PET scan for sinonasal malignancies. This is highlighted by the latest international consensus statement on endoscopic skull base surgery.

**Objective:**

This systematic review aims to clarify the role of PET scan in the management of sinonasal malignancies.

**Methods:**

We conducted a comprehensive literature search using PubMed, MEDLINE, EMBASE, Web of Science, CINAHL, and Cochrane databases for research studies of interest. The Preferred Reporting Items for Systematic Reviews and Meta-analyses (PRISMA) updated statement was used to guide the review.

**Results:**

In total, 1807 articles were assessed for eligibility. Thirty-nine original papers, published between 2004 and 2021, met inclusion criteria. Seven articles focused on the role of PET scan for inverted papilloma, 23 for sinonasal carcinoma, 4 for melanoma, and 3 for lymphoma, and finally, 3 articles focused on the use of specific PET scan tracers for sinonasal malignancies. Qualitative summaries for each potential role of PET scans were provided. In general, included studies were retrospective in nature with low level of evidence.

**Conclusions:**

In general, and across all types of sinonasal malignancies, PET scan yielded positive results regarding detection and initial staging. It was also considered as the modality of choice for detection of distant metastases, except in the case of sinonasal lymphoma. PET scan's main limit resides in its inability to detect lesions in or close to the metabolic activity of the brain.

## Introduction

A positron emission tomography (PET) scan is a type of medical imaging that allows visualization of metabolic or biochemical function of underlying tissues and organs.^1^ A tracer is used, commonly fluorodeoxyglucose, to demonstrate metabolic activity.^1^ To quantify the degree of metabolic activity, the standardized uptake value (SUV) is measured.^2^ Several types of SUV may be reported: the SUV_mean_ is the average SUV in the region of interest, and the SUV_max_ is the highest SUV value found.^3^ In order to improve imaging resolution, a PET scan can be combined with another anatomic imaging modality, such as computed tomography (CT) or magnetic resonance imaging (MRI), and is then called PET-CT or PET-MRI. Neoplastic cells have a higher metabolic rate than non-neoplastic cells. Therefore, in oncologic cases, a PET scan offers substantial advantages over traditional anatomic imaging, such as CT or MRI, allowing for detection of new or recurrent cancers.^1^ It can also help distinguish benign from malignant lesions.^1^

In head and neck oncology, PET-CT has multiple roles including detection of unknown primaries and assessment of distant metastases in advanced cancers.^4^ However, even though PET-CT has drastically changed head and neck imaging and management, it is still subject to a variety of interpretation pitfalls, including physiological fluorodeoxyglucose (FDG) uptake in the head and neck, the presence of benign lesions, or inflammation and scarring due to recent surgery.^5^ Therefore, PET-CT results must be correlated with clinical information and, if needed, other imaging modalities, such as MRI or ultrasound.^5^

Despite its wide adoption for cancers of the oral cavity, oropharynx, larynx, and hypopharynx, the role of PET scan is not well understood for the management of sinonasal malignancies, where normal metabolic activity of the brain may interfere with detection. Currently, CT and MRI are the main imaging tools for sinonasal malignancies, assessing size, nature, extent, and invasion of said tumor.^6^ Contrast-enhanced CT is also used for diagnosis of hypervascular tumors.^6^ CT and MRI lack however the metabolic information provided by a PET scan, which may be a useful addition for tumor evaluation.^7^ Although an international consensus statement on the management of sinonasal malignancies and anterior skull base tumors was published in 2019, the role of PET scan was not discussed.^8^ As a result, there is still a lack of consensus on the role of PET scans in the management of sinonasal malignancies, justifying a systematic review.

The objective of this systematic review is to discuss the potential role of PET scan in the management of sinonasal malignancies.

## Materials and Methods

A systematic review was designed and conducted based on the Preferred Reporting Items for Systematic Reviews and Meta-analyses (PRISMA) updated statement.^9^ This involved a multi-step process as outlined below. The study has not been registered. Institutional review board (IRB) approval was not necessary, as this was a systematic review.

### Search Strategy

Two independent reviewers searched the literature to retrieve all relevant studies published before November 25, 2022. The search was performed in PubMed, CINAHL, Cochrane, EMBASE, Web of Science, and Medline. Different search terms were used according to the database to optimize the pertinence and number of articles found. For PubMed, Cochrane, Web of Science, and Medline, Medical Subject Headings (MeSH) were used. The search string was as follows: “(‘Skull Base’[Mesh] OR sinonasal* OR sinus*) AND (‘Neoplasms’[Mesh] OR malignancy* OR tumor*) AND (‘Positron-Emission Tomography’[Mesh] OR pet scan* OR fdg-pet scan*).” For CINAHL, we used the search string: “Sinonasal Malignancy AND PET scan.” For EMBASE, we used the search string: “(‘sinonasal’ OR ‘sinus’) AND (‘Neoplasm’ OR ‘malignancy’) AND (‘Positron-Emission Tomography’ OR ‘pet scan’ OR ‘fdg scan’).” The PRISMA criteria were followed.^9^

### Eligible Criteria

The inclusion criteria were articles in English, retrospective or prospective in nature, with a primary objective of discussing PET scan for sinonasal malignancies, and conducted in a human population. The exclusion criteria were articles not in English, case reports, commentaries, editorials, and studies that did not assess the use of PET scan in sinonasal malignancies or that were conducted in animals.

### Selection Process

All articles found across the databases using the search terms were pooled together, and duplicates were recorded and removed manually. Two independent reviewers then performed a first round of title and abstract screening. Articles that were initially included were then assessed based on the full-text using inclusion and exclusion criteria. Conflicts were resolved by discussion.

### Outcomes of Interest

Selected articles were grouped by themes and objectives to summarize the results. The qualitative and quantitative data from the articles were extracted by the 2 reviewers. Data such as SUV_mean_ and SUV_max_ were collected where possible, in addition to sensitivity and specificity of PET scan for sinonasal malignancies in preoperative and postoperative periods. A qualitative outcome description has been performed and displayed in tables, in which each study has a dedicated column. The risk of bias of individual studies was assessed using the Robin-I assessment tool, while the level of evidence was assessed by both reviewers using the Oxford Centre for Evidence-Based Medicine: Levels of Evidence.^10^ Two reviewers (DE and NY) independently analyzed the quality of selected papers. Any disagreement was solved by discussion.

## Results

**Figure 1.**
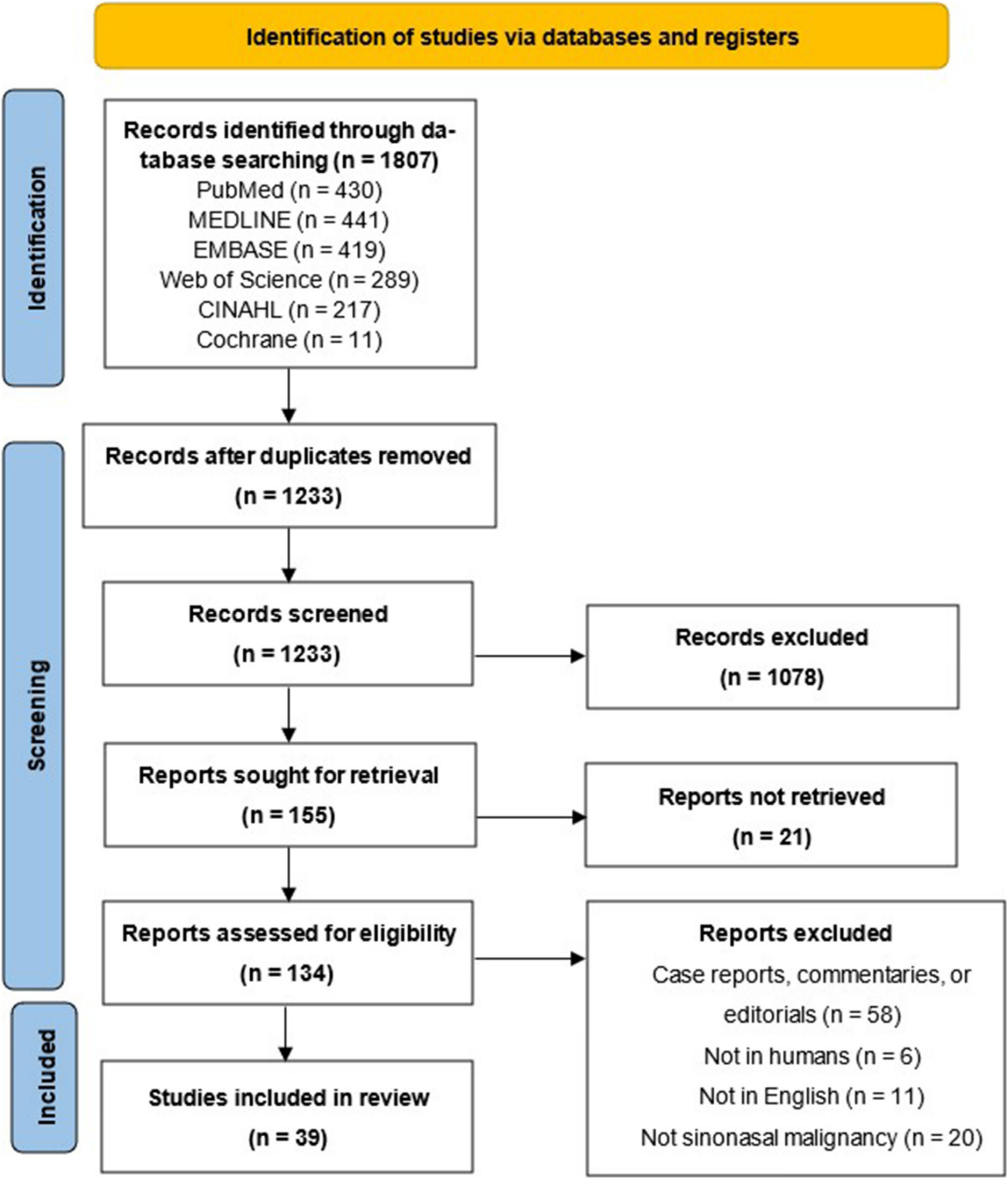
Preferred reporting items for systematic reviews and meta-analyses guidelines (PRISMA) flow diagram.

A total of 1807 articles were identified using our search strategies across all databases. After eliminating all duplicates, 1233 articles were screened with titles and abstracts, and 134 articles with the full text. In the end, 39 articles met inclusion criteria and were included in the systematic review. The PRISMA flow diagram may be found in Figure 1.

### Inverted Papilloma and Squamous Cell Carcinoma

Sinonasal papillomas are benign tumors of epithelial origin that occur in several histologic forms: inverted, cylindrical, and fungiform.^11^ Inverted papillomas (IP) account for 47% of all sinonasal papillomas and are locally aggressive. Given that they may harbor squamous cell carcinoma (SCC) in 2% to 53%, differentiating IPs without and with SCC is of clinical interest to determine the optimal management plan preoperatively.^11–14^ Although some studies suggest that the use of MRI may be useful in identifying IPs with SCC through the loss of convoluted cerebriform pattern, which is characteristic of IPs, diagnosis is still often made after resection with histopathology.^15^

Since PET scans are powerful diagnostic tools for the diagnosis, staging, and surveillance of malignancies, studies have been performed to investigate its utility in differentiating and managing IPs without and with SCC.

A total of 7 studies discussing the use of PET scan in the management of IP were included.^16–22^ A summary of each study, their level of evidence, and an assessment of risk of bias can be found in Table 1. Six of the 7 studies were retrospective case series or case-control studies with serious risk of bias, while 1 study was a prospective non-consecutive cohort study with moderate risk of bias.

**Table 1.** Summary of Included Studies Discussing the Use of PET Scan for Inverted Papilloma.

Author (year)	Study objective	Imaging modality	Sample size	SUV_max_ (range) SUV_mean_ (range)	Findings	Type of study (level of evidence)	Bias assessment
Ninomiya (2004)	Describe findings of sinonasal tumors using FDG and choline PET	FDG-PET ^11^C-PET	5 IP without SCC	n/a 2.0-4.7 n/a 2.3-5.2	No statistical difference in SUV between IP and malignancies with FDG, but difference with ^11^C	Retrospective 4	Serious
Shojaku (2007)	Compare findings of IP without and with SCC	FDG-PET	3 IP without SCC 2 IP with SCC	4.9-7.3 n/a 8.9-20.9 n/a	Both IP with and without SCC have high SUV	Retrospective 4	Serious
Jeon (2009)	Investigate findings of IP without and with SCC	FDG-PET/CT	2 IP without SCC 6 IP with SCC	6.2-7.8 n/a 13.3-31.9 n/a	High SUV does not necessarily imply presence of SCC	Retrospective 4	Serious
Cohen (2009)	Describe findings of sinonasal papillomas	FDG-PET/CT	4 without SCC 1 cylindrical papilloma without SCC	3.5-9.0 2.2-6.2 43.0 30.0	High SUV may be present in benign papilloma	Retrospective 4	Serious
Allegra (2010)	Role of PET in surveillance of IP	FDG-PET	2 IP without SCC 1 IP with SCC	4.5-6.1 n/a 8.1 n/a	Detection of recurrence in all cases	Retrospective 4	Serious
Yilmaz (2015)	Determine if there is a difference in FDG uptake between IP and malignancies	FDG-PET/CT	10 IP without SCC 9 SCC	2.5-31.0 n/a 6.1-37.8 n/a	Statistical difference in SUV_max_ between IP and SCC. Use however clinical judgment	Prospective 3b	Moderate
Tzelnick (2018)	Describe implications of incidental sinonasal FDG uptake	FDG-PET/CT	4 IP without SCC	3.5-5.2 2.0-3.6	SUV values of IP when incidentally found	Retrospective 4	Serious

Abbreviations: IP, inverted papilloma; SCC, squamous cell carcinoma.

Of the 7 studies, 6 focused on describing the characteristics of IPs without SCC and, when possible, comparing them to IPs with SCC or sinonasal SCC. The main parameter which was studied was FDG avidity. Although most studies had 3 to 8 patients, 1 included 19 patients. In general, both benign IPs and IPs with SCC had high SUV values. Statistical comparisons in SUV values were performed in 2 studies, where 1 study did not find a statistical difference in SUV values, while 1 did. However, both studies in which statistical comparisons were performed were done between patients with benign IPs and sinonasal malignancies, not IPs with SCC. Although most studies describe higher SUV values in patients with IP harboring SCC compared to benign IP, there is still an overlap in values. SUV values of over 30 have been reported in benign papillomas. However, this is often in association with IPs also harboring a component of oncocytic or cylindrical papilloma.^19,21^

Interestingly, 1 study examined whether the pattern of FDG avidity in benign IPs and IPs with SCC was different.^15^ However, both benign IPs and IPs with SCC could have homogeneous and heterogeneous uptake.

Of the 7 included studies, 1 examined the role of the PET scan in surveillance of IP recurrence after surgical resection.^20^ Although the series was limited to 4 patients, only 3 had histology-proven recurrence. All 3 recurrences were detected by the exam.

**Take-home points:** Most authors are cautious in stating that PET scans can help differentiate benign IPs from IPs harboring SCC and other sinonasal malignancies. Statistical comparisons between IPs and sinonasal carcinomas were unable to show a clear differentiation on the sole basis of SUV values. However, PET/CT was able to detect all recurrences of IP after surgical resection.

### Sinonasal Carcinomas

Five potential roles of PET scans for the management of sinonasal malignancies were identified across all included studies: detection and initial staging, characterization of FDG avidity with SUV values, prognosis, treatment planning, and surveillance. Each of these potential roles is discussed separately below.

#### Detection and Initial Staging

A total of 5 studies discussing the diagnostic performance indices of the PET scan for the detection and initial staging of sinonasal malignancies were included.^23–27^ A summary of each study, their level of evidence, and an assessment of risk of bias can be found in Table 2. Four of the 5 studies were retrospective case series with serious risk of bias, while 1 study was a prospective non-consecutive cohort study with moderate risk of bias.

**Table 2.** Summary of Included Studies Discussing the Performance Indices of PET Scans for Staging.

Author (year)	Study objective	Imaging modality	Sample size	Findings	Type of study (level of evidence)	Bias assessment
Gil (2007)	Describe the role of PET scans for preoperative staging and postoperative surveillance of skull base tumors	FDG-PET/CT	17 SCC 9 Sarcoma 4 ACC 4 ENB 2 MPNST 2 SNUC 1 Hemangioma 1 Chordoma 1 Clear cell carcinoma 1 Hemangiopericytoma 1 JNA 1 Meningioma 2 Adenocarcinoma 1 NPC	Preoperative detection sensitivity for primary tumor: 100% for all tumor types, except ACC (80%) ENB (66%) MPNST (50%) JNA (0%) Overall pre- and postoperative primary tumor detection: Sensitivity: 77% Specificity: 81% PPV: 83% NPV: 76%	Prospective 3b	Moderate
Lamarre (2011)	Describe the role of PET scans for the initial staging and posttreatment surveillance of paranasal sinus carcinomas	FDG-PET/CT	9 ENB 6 SCC 6 SNUC 6 Melanoma 4 Salivary gland carcinoma	Initial primary tumor and neck and distant metastasis detection: Sensitivity: 100%, n/a, 100% Specificity: 100%, 92%, 92% PPV: 100%, n/a, 50% NPV: 100%, 100%, 100%	Retrospective 4	Serious
Ramakrishnan (2013)	To determine if PET scan accurately diagnoses and stages sinonasal malignancies	FDG-PET or FDG-PET/CT	19 SCC 7 SNUC 7 Melanoma 6 Salivary gland carcinoma 5 ENB 3 Sarcoma 1 Clear cell carcinoma 1 Adenocarcinoma 1 Plasmacytoma	Initial primary tumor detection: Sensitivity: 94%	Retrospective 4	Serious
Hines (2016)	To determine the PPV and NPV of PET scans in determining malignancy in skull base lesions and perform a systematic review for optimal PET interpretation	FDG-PET/CT	5 SNUC 3 Melanoma 2 NPC 2 Metastasis of renal cell carcinoma 1 Adenocarcinoma 1 IgG4 disease 1 SCC	Overall pre- and posttreatment detection of malignancy using radiologic interpretation, SUV ≥ 2.5 and SUV ≥ 3.0: Sensitivity: 100%, 94%, 81% Specificity: 73%, 33%, 60% PPV: 80%, 60%, 68% NPV: 100%, 83%, 75%	Retrospective 4	Serious
Meerwein (2020)	To assess PET imaging for initial staging of sinonasal malignancies	FDG-PET/CT or FDG-PET/MRI	28 Melanoma 12 SCC 9 SNUC 8 Adenocarcinoma 5 ACC 3 ENB	Initial primary tumor and neck and distant metastasis detection: Sensitivity: 95%, 100%, 100% Specificity: n/a, 92%, 98% PPV: n/a, 50%, 87% NPV: n/a, 100%, 100%	Retrospective 4	Serious

Abbreviations: SCC, squamous cell carcinoma; ACC, adenoid cystic carcinoma; ENB, esthesioneuroblastoma; MPNST, malignant peripheral nerve sheath tumor; SNUC, sinonasal undifferentiated carcinoma; JNA, juvenile nasopharyngeal angiofibroma; NPC, nasopharyngeal carcinoma; PPV, positive predictive value; NPV, negative predictive value.

Reporting of diagnostic performance indices varied significantly between studies, making it difficult to pool results quantitatively. All studies commented on the sensitivity of PET scans in detecting primary sinonasal and skull base tumors. Since most studies were case series which included biopsy proven sinonasal tumors, specificity for primary tumor assessment often could not be reported. Nonetheless, sensitivity was generally good with values varying between 77% and 100%, while specificity values were generally lower between 73% and 100%. In the study by Gil et al, a sensitivity of 0% was reported for juvenile nasopharyngeal angiofibroma (JNA), because the disease in the 1 patient with JNA could not be detected by PET/CT.^23^

Given that there is still uncertainty regarding a standardized SUV cut-off value indicative of malignant lesions for skull base tumors, all studies relied on the interpretation of the radiologist, nuclear medicine specialist, and surgeon to determine if a sinonasal lesion was malignant on PET scans. One study used an SUV value of 2.5 to indicate whether a lesion was suspicious, and positivity was determined according to the interpretation of the physician.^25^ Another study compared the diagnostic performance indices of PET scans when using SUV cut-off values of 2.5 and 3.0 and when solely using the interpretation of the radiologist.^26^ This study also included a systematic review to determine if there was an ideal SUV cut-off value described in the literature. Conclusions from the study were that there is currently no consensus for an ideal SUV value to determine whether a skull base lesion is malignant.

Additionally, the clinical impact of using PET scans on the initial staging of sinonasal malignancies has been investigated in 3 studies that were included in the systematic review.^23,28,29^ The first was a retrospective study of a non-consecutive cohort, while the second was a retrospective study of a consecutive cohort.^28,29^ The last study used a prospective design.^23^ Bias assessment revealed serious risk for the first study and moderate for the last 2.

In the study by Maurer et al, 46 hybrid FDG PET/CT or PET/MRI were performed for the initial staging of sinonasal malignancies in addition to conventional CT scan and MRI. Additional radiologic information for the primary tumor was obtained in 4.3% of the cases, for regional neck metastasis in 19.1%, for distant metastasis in 10.6%, and for second primary tumor in 4.3%. Hybrid FDG PET/CT or PET/MRI did not reveal any additional clinically relevant information for the primary tumor such as orbital or dural involvement or for regional neck metastasis. In the study by Wild et al, 9 FDG PET/CT scans were performed for consecutive patients with sinonasal malignancies in addition to CT scan or MRI. For the primary tumor, although additional radiologic information was obtained for 1 patient out of 9 with the FDG PET/CT scan, this did not affect the treatment plan. However, for distant metastasis and second primary tumor assessment, 2 patients out of 9 had their treatment plan changed with additional clinically relevant information. Lastly, in the study by Gil et al, out of 47 patients who prospectively had FDG PET/CT scans in addition to conventional imaging for staging or re-staging of sinonasal malignancies, performing the FDG PET/CT accurately upstaged 1 patient preoperatively and 10 patients postoperatively in the surveillance period. Based on these findings, all 3 groups of authors concluded that the use of PET/CT could be useful to correctly stage patients with sinonasal malignancies.

Finally, perhaps the most clinically relevant point raised in this study is the difficulty for PET scan to detect lesions close to the brain. Indeed, 5 out of 7 studies highlighted the impact of physiologic FDG uptake of the brain and stated that it greatly hampered the ability of PET scan to detect primary lesions and that it could even mask intracranial primary tumor extensions from the paranasal sinuses.^23–26,29^

**Take-home points:** PET/CT has good sensitivity and specificity for initial detection of primary tumors, neck, and distant metastases. The added usefulness of PET scans in the initial staging of sinonasal malignancies mostly appears to be on the detection of distant metastasis and second primary tumors, rather than adding clinically relevant information on the primary tumor. Lesions close to or in the cerebral cavity could not be detected.

#### Characterization of FDG Avidity With SUV Values

Six studies describing the FDG avidity of sinonasal malignancies according to histopathology were identified.^16,22,30–33^ All studies were retrospective in nature. Reporting of FDG avidity for each type of tumor varied between studies. While some studies reported individual values of SUV for each case of sinonasal malignancy, other studies reported average SUV values. Furthermore, some studies only reported SUV_max_ values, while others reported SUV_mean_ values. One study did not report any quantitative values and visually categorized FDG avidity into mild and avid uptake. A summary of each study and reported values of FDG avidity for different types of sinonasal malignancies can be found in Table 3.

**Table 3.** SUV Values for Sinonasal Malignancies According to Histopathology.

Author (year)	Squamous cell carcinoma SUV_max_ (range) SUV_mean_ (range)	Adenocarcinoma SUV_max_ (range) SUV_mean_ (range)	Malignant melanoma SUV_max_ (range) SUV_mean_ (range)	Sinonasal undifferentiated carcinoma SUV_max_ (range) SUV_mean_ (range)	Adenoid cystic carcinoma SUV_max_ (range) SUV_mean_ (range)	Olfactory neuroblastoma SUV_max_ (range) SUV_mean_ (range)	Neuroendocrine carcinoma SUV_max_ (range) SUV_mean_ (range)
Ninomiya (2004)	n/a 5.7-8.8	n/a 2.8-6.0	n/a 5.5	−	−	−	−
Felix-Ravelo (2016)	−	2.7-20.3 n/a	2.6-10.9 n/a	7.6-24.0 n/a	3.8-9.8 n/a	5.0-8.7 n/a	2.8-7.7 n/a
Fujima (2016)	No range reported Mean SUV_max_ (SD): 19.9 (6.9) Mean SUV_mean_ (SD): 9.9 (3.6)	−	−	−	−	−	−
Elkhatib (2016)	−	−	−	12.1-52.9 n/a	−	4.6-10.7 n/a	−
Tzelnick (2019)	4.5-26.1 2.4-15.7	8.0 5.7	−	−	−	−	4.7 3.5
Ozturk (2020)	No SUV values Mild uptake: 0% Avid uptake: 100%	−	No SUV values Mild uptake: 0% Avid uptake: 100%	No SUV values Mild uptake: 0% Avid uptake: 100%	No SUV values Mild uptake: 0% Avid uptake: 100%	No SUV values Mild uptake: 13% Avid uptake: 87%	−

Abbreviation: SD, standard deviation.

**Take-home points:** Esthesioneuroblastoma (ENB), malignant peripheral nerve sheath tumor (MPNST), and JNA may present with lower SUV values on average; SUV values for ENB range from 4.6 to 10.7, while sinonasal undifferentiated carcinoma (SNUC) and SCC may present with higher SUV values, respectively, 7.6 to 52.9 and 2.4 to 26.1.

#### Prognosis

A total of 7 studies were found discussing the role of PET scan for prognosis in sinonasal malignancies.^27,34–39^ A summary of each study, their level of evidence, and an assessment of risk of bias can be found in Table 4. All 7 studies were retrospective and had a serious risk of bias.

**Table 4.** Summary of Included Studies Discussing the Value of PET Scans for Prognosis.

Author (year)	Study objective	Imaging modality	Sample size	Findings	Type of study (level of evidence)	Bias assessment
Rho (2009)	Investigate the role of PET scans in detection and prediction of local recurrences of maxillary sinus cancer	FDG-PET/CT	22 SCC	Visual assessment is the most potent predictor for prediction of local recurrence of maxillary sinus cancer: PPV: 85.7% NPV: 94.1%	Retrospective 4	Serious
Seol (2013)	Assess whether SUV_max_ can be used to predict survival in patients with paranasal sinus malignancy	FDG-PET	38 SCC	High SUV_max_ group had poorer outcome than low SUV_max_ group irrespectively of tumor staging	Retrospective 4	Serious
Doi (2016)	Determine whether SUV_max_ of primary lesion has prognostic value in maxillary sinus cancer	FDG-PET/CT	29 SCC 2 non-SCC	High SUV_max_ for primary lesion results in higher risk of cancer recurrence and death	Retrospective 4	Serious
Kim (2016)	Investigate the value of parameters assessed with FDG-PET/CT in predicting recurrence and survival in patients with nasal cavity and paranasal sinus cancer	FDG-PET/CT	31 SCC 2 ACC 2 Adenocarcinoma 2 Sarcoma 1 Small cell carcinoma	Tumoral heterogeneity and volumetric parameters of FDG PET are important independent prognostic predictors	Retrospective 4	Serious
Doi (2018)	Assess the feasibility of FDG-PET volumetric parameters to predict prognosis of MSSCC before undergoing IA-CRT	FDG-PET/CT	24 SCC	FDG-PET parameters show no significant predictive information	Retrospective 4	Serious
Abu-Ghanem (2018)	Describe the prognostic value of the first posttreatment whole-body PET/CT in patients with sinonasal and skull base malignancies	FDG-PET/CT	9 SCC 6 Melanoma 5 ACC 5 Adenocarcinoma 4 ENB 2 SCT 2 SNUC 2 Metastasis 1 Sarcoma 1 MPNST	First posttreatment PET/CT for prediction of persistent/recurrent disease and survival at the end of follow-up period: Sensitivity: 85.7% Specificity: 87.5% PPV: 80% NPV: 91.3%	Retrospective 4	Serious
Meerwein (2020)	Assess PET scan in initial staging and outcome prediction of sinonasal malignancies	FDG-PET/CT	28 Melanoma 12 SCC 9 SNUC 8 Adenocarcinoma 5 ACC 3 ENB	TLG of primary tumor is an independent prognostic factor for achieving complete remission after treatment	Retrospective 4	Serious

Abbreviations: MSSCC, maxillary sinus squamous cell carcinoma; IA-CRT, intra-arterial infusion chemoradiotherapy; SCT, spindle cell tumor; MPNST, malignant peripheral nerve sheath tumor; TLG, total lesion glycolysis.

Most included studies discussed prognosis of sinonasal malignancies using parameters such as disease-specific survival (DSS) and recurrence-free survival (RFS). Two out of 7 studies used the SUV_max_ of the primary tumor to predict survival in patients. Both studies had similar outcomes, where a high SUV_max_ was correlated with a poorer outcome, for disease recurrence and overall survival. Interestingly, in the first study, patients with high SUV_max_ had a significantly worse prognostic regardless of T staging, whereas patients with low SUV_max_ had a better prognosis when T staging was between T1 and T3.^35^ In the second study, when using a PET scan for the assessment of the primary lesion, a higher SUV_max_ was related to a higher risk of cancer recurrence and death.^36^

Several PET scan parameters have been previously assessed for prognostic value in a variety of tumors.^40^ As previously reported, SUV is the most widely used PET parameter, notably due to its easy access with commercial workstations.^40^ Nonetheless, other parameters also exist. Volumetric parameters such as metabolic tumor volume (MTV) and total lesion glycolysis (TLG) are measured after tumor delineation and are known to have prognostic values.^40^ While MTV mostly weights the volumetric burden of the tumor, TLG, being the product of MTV and SUV_mean_, weights both volumetric burden and metabolic activity of tumors.^40^

In our research for sinonasal malignancies, 2 studies found that TLG was the superior PET/CT parameter in respect to disease survival. One study cited tumor heterogeneity to be a prognostic factor for disease recurrence. However, a third study found that no parameters on FDG-PET/CT had significant predictive information.

**Take-home points:** Higher SUV values were associated with poorer outcomes, regardless of conventional staging, while malignancies with lower SUV values had a different outcome depending on conventional staging.

#### Treatment

The role of PET/CT scans has been investigated for radiotherapy treatment planning in head and neck cancers. Although evidence supports that PET/CT may impact planning by changing the accuracy and delineation of gross tumor volumes, only 0% to 19% of the patients in those cohorts have sinonasal malignancies.^41–44^ No study specifically investigating the role of PET scans for radiotherapy treatment planning in patients with sinonasal malignancies has been performed. Thus, further investigations should be performed before any conclusions can be drawn.

#### Surveillance

A total of 4 studies discussing the role of PET scan for surveillance of sinonasal malignancies were included.^23,24,45,46^ A summary of each study, their level of evidence, and an assessment of risk of bias can be found in Table 5. Three out of 4 studies were retrospective case series studies with serious risk of bias, while 1 was a prospective non-consecutive cohort study with moderate risk of bias.

**Table 5.** Summary of Included Studies Discussing the Value of PET Scans for Surveillance.

Author (year)	Study objective	Imaging modality	Sample size	Findings	Type of study (level of evidence)	Bias assessment
Gil (2007)	Describe the role of PET scans for preoperative staging and postoperative surveillance of skull base tumors	FDG-PET/CT	17 SCC 9 Sarcoma 4 ACC 4 ENB 2 MPNST 2 SNUC 1 Hemangioma 1 Chordoma 1 Clear cell carcinoma 1 Hemangiopericytoma 1 JNA 1 Meningioma 2 Adenocarcinoma 1 NPC	Postoperative follow-up using PET/CT enables early detection of tumor recurrence	Prospective 3b	Moderate
Lamarre (2011)	Describe the role of PET scans for the initial staging and posttreatment surveillance of paranasal sinus carcinomas	FDG-PET/CT	9 ENB 6 SCC 6 SNUC 6 Melanoma 4 Salivary gland carcinoma	Primary, neck, and distant restaging: Sensitivity: 77%, 100%, 83% Specificity: 84%, 89%, 95% PPV: 56%, 54%, 63% NPV: 93%, 100%, 98%	Retrospective 4	Serious
Workman (2017)	Assess the value of PET scan as a screening tool for surveillance in asymptomatic patients	FDG-PET/CT	14 ENB 6 SCC 6 Poorly differentiated carcinoma 5 SNUC/SNEC 4 Adenocarcinoma 4 Melanoma 3 Spindle cell carcinoma 2 Sarcoma 1 Ex pleomorphic adenoma	Surveillance diagnostic performance indices: Sensitivity: 75% Specificity: 96.3% PPV: 42.9% NPV: 99%	Retrospective 4	Serious
Ozturk (2019)	Determine the utility of posttreatment whole-body PET/CT in surveillance for sinonasal malignancies	FDG-PET/CT	30 SCC 7 ENB 3 Adenocarcinoma 5 SNUC 2 Small cell carcinoma 8 Melanoma 6 Rhabdomyosarcoma 4 Poorly differentiated carcinoma 8 ACC 2 Osteosarcoma 2 Neuroendocrine carcinoma 2 Carcinosarcoma 1 Clear cell carcinoma	Local recurrence, regional nodal metastasis, distant metastasis: Sensitivity: 84%, 91%, 81% Specificity: 95%, 99%, 99% PPV: 84%, 91%, 97% NPV: 95%, 99%, 96%	Retrospective 4	Serious

Abbreviations: SCC, squamous cell carcinoma; ACC, adenoid cystic carcinoma; ENB, esthesioneuroblastoma; MPNST, malignant peripheral nerve sheath tumor; SNUC, sinonasal undifferentiated carcinoma; SNEC, small cell neuroendocrine carcinoma; JNA, juvenile nasopharyngeal angiofibroma; NPC, nasopharyngeal carcinoma; PPV, positive predictive value; NPV, negative predictive value.

All 4 studies assessed the value of PET/CT in posttreatment surveillance for patients with sinonasal malignancies and concluded that PET/CT is a valuable tool for posttreatment detection of locoregional recurrences and distant metastases.

However, the optimal timing for posttreatment PET/CT differs from 1 study to another. One study recommends waiting 1 month after completion of treatment to decrease the risk of false negative readings, while another study recommends waiting 6 months for optimal reading.^45,46^

Two out of 4 studies also highlighted the inability of PET scan to accurately detect brain metastases on follow-up, due to the normal metabolic activity of the brain.^23,24^ MRI was required for detection.^24^

**Take-home points:** PET/CT can be used for posttreatment detection of locoregional recurrences and distant metastases. However, it was unable to detect brain metastases on follow-up.

### Sinonasal Melanoma

Mucosal malignant melanoma (MMM) is a very rare tumor accounting for around 1.3% of all melanomas.^47^ Most MMMs (55.4%) arise from the head and neck region, while other sites, such as the female genitalia, the anus and rectum, and the urinary system, may also be affected.^47^ Sinonasal malignant melanomas account for 4% of all head and neck malignant melanomas.^48^

PET scan is an effective and accepted imaging modality for staging of cutaneous malignant melanoma and for screening of distant metastases.^49^ However, because of the rare incidence of sinonasal melanoma, there are few studies investigating the role of PET scans for MMM.

A total of 4 studies discussing the role of PET scan in the management of sinonasal melanoma were included.^50–53^ A summary of each study, their level of evidence, and an assessment of risk of bias can be found in Table 6. All 4 studies were retrospective case series with serious risk of bias.

**Table 6.** Summary of Included Studies Discussing the Use of PET Scan for Sinonasal Melanoma.

Author (year)	Study objective	Imaging modality	Sample size	SUV_max_ (range) SUV_mean_ (range)	Findings	Type of study (level of evidence)	Bias assessment
Goerres (2002)	Describe findings of sinonasal melanomas using FDG-PET	FDG-PET	10 MMM	n/a	FDG-PET can detect melanoma in addition to locoregional and distant metastasis	Retrospective 4	Serious
Haerle (2011)	Compare findings on PET/CT with CT, MRI, or PET alone in MMM	FDG-PET/CT	10 MMM	3.4-11.5 n/a	PET/CT superior to CT, MRI, or PET alone for initial staging and follow-up in MMM	Retrospective 4	Serious
Inubushi (2012)	Investigate the prognostic value of FLT-PET/CT for MMM undergoing CIRT	FLT-PET/CT	13 MMM	2.99-9.76 n/a	Higher pre-CIRT SUV suggests better overall and metastasis-free survival	Retrospective 4	Serious
Samstein (2016)	Evaluate association between PET/CT findings and survival	FDG-PET/CT	78 MMM	n/a	Lower postradiotherapy FDG uptake suggests better survival	Retrospective 4	Serious

Most studies included 10 to 13 patients, while 1 included 78 patients. Three out of 4 studies described findings of sinonasal melanoma using FDG-PET scan or FDG-PET/CT, and of these, 1 compared findings on PET/CT with CT, MRI, or PET scan alone. In these studies, PET scan or PET/CT was able to find all primary tumors, which all presented with increased FDG uptake.^50,51,53^ The size, shape, and location of the lesion was important for visibility of tumor and FDG uptake. Tumors with a diameter >3 cm and with a nodular appearance were more visible on PET scan. Lesions in the anterior part of the nose were harder to assess because of the presence of muscles and other non-specific elements raising FDG uptake. Lesions were more visible in the posterior part of the nose where muscle uptake is unlikely. Combining PET scan and CT was considered superior to PET scan, CT, or MRI alone due to additional anatomic and metabolic information for staging, to distinguish scar tissue from melanoma masses, and to give exact spatial resolution for surgical planning.^51^ Contrast-enhanced CT with PET scan was deemed better for adequate staging and restaging.^51^ PET scan or PET/CT could also detect locoregional recurrences and distant metastases on follow-up and was deemed to be the modality of choice for this matter.

As part of the limitations of PET scan, 2 studies highlighted the inability of this imaging modality to differentiate postsurgical inflammation from local recurrences at the site of the primary tumor or from inflammation such as sinusitis. Moreover, PET/CT was unable to find cerebral metastases in a case of MMM due to high background metabolism in the intracranial cavity, and MRI was needed for proper visualization. Finally, smaller lesions <1 cm were hardly visible on PET scan alone but easily visible on PET/CT.

One study interestingly evaluated the efficacy of fluorothymidine (FLT)-PET/CT to determine prognostic value of SUV_max_ for patients with MMM undergoing carbon ion radiotherapy (CIRT).^52^ Results suggest a higher SUV_max_ pre-CIRT has a better overall survival in addition to having better metastasis-free survival for patient prognosis. Another study found no significant association between preoperative and postoperative SUV values with conventional FDG-PET scans regarding prediction of disease recurrence or overall survival.^53^ However, it did find that overall survival, DSS, and locoregional RFS was better when postradiotherapy SUV_max_ was less than 4, improving median DSS from 14 months to 56 months.

**Take-home points:** PET scan was a good tool to detect primary melanomas in the sinonasal region, and used in combination with CT, it was deemed the superior modality over PET, MRI, or CT alone. PET/CT was also good in detecting locoregional recurrences and distant metastases on follow-up. Once again, PET was unable to detect brain metastases.

### Sinonasal Lymphoma

Lymphoma accounts for 5% of all head and neck malignancies, and the nasal cavity and paranasal sinuses are affected in 0.2% to 2% of all cases of lymphomas.^54^ Incidence and immunophenotypic characteristics of the disease are highly variable depending of geographical areas.^55^ Sinonasal lymphomas (SLs) with natural killer (NK) and T-cell phenotypes are more common in Far East Asian countries, in Mexico, and in South America, while B-cell lymphoma is more common in Western populations.^55^ Patients with SLs are often positive for Epstein–Barr virus (EBV), in more than 80% of cases.^56^

PET scans are commonly used in patients with lymphoma.^57^ It is reported that the use of PET scan leads to a change in staging in up to 20% to 40% of patients, while it changes the treatment choice in 5% to 15% of patients.^57^ Remission documentation using PET scan is also common practice for patients with lymphoma.^57^ However, the value of PET scan in surveillance of posttreatment relapse is still limited.^57^

SL is a rare disease, and only a few articles have been published discussing the role of PET scan imaging for this disease entity. A total of 3 articles discussing this potential role were included.^54,56,58^ A summary of each study, their level of evidence, and an assessment of risk of bias can be found in Table 7. All 3 studies were retrospective case series with serious risk of bias.

**Table 7.** Summary of Included Studies Discussing the Use of PET Scan for Sinonasal Lymphoma.

Author (year)	Study objective	Imaging modality	Sample size	SUV_max_ (range) SUV_mean_ (range)	Findings	Type of study (level of evidence)	Bias assessment
Karantanis (2008)	Assess the value of PET/CT in sinonasal NK/T-cell lymphoma	FDG-PET/CT FDG-PET	10 NK/T-cell lymphoma	4.6-25.0 n/a	PET/CT is a valuable complementary tool for assessing extent of disease and monitoring therapy response	Retrospective 4	Serious
Tempescul (2009)	Describe findings of primary NHLs of the sinonasal tract using PET/CT	FDG-PET/CT	2 B-cell lymphoma	3.3-5.9 n/a	PET/CT useful for initial staging	Retrospective 4	Serious
Kim (2018)	Compare SUV_max_ values of SL and SCC	FDG-PET/CT	39 non-Hodgkin’s lymphoma 39 SCC	10.6-16.4 n/a 11.6-18.7 n/a	No significant difference between SUV_max_ of NHL and SCC	Retrospective 4	Serious

The 3 included studies contained respectively 10, 2, and 78 patients. The 2 first studies described the value of PET/CT for the management of SL.^54,56^ PET/CT was deemed to be an excellent tool for initial staging of SL, the first article assessing NK/T-cell lymphoma and the second assessing B-cell lymphoma. In fact, disease in the nasal cavity and paranasal sinuses was intensely avid for FDG uptake, with an SUV_max_ ranging from 5 to 25.^56^

Regarding therapy response assessment, PET/CT was also remarkably useful, mostly for nasal disease, where all findings were consistent with histopathology. Successfully treated lymphoma had the same uptake as the surrounding soft tissue, even though at initial staging it was intensely avid. Persistent disease was also easily distinguishable and had high enough FDG uptake to differentiate it from low-grade inflammatory uptake. However, for extranasal disease, PET/CT was a good tool to detect lymph node, skin, and focal bone lesions, but repeatedly was unable to detect diffuse bone marrow involvement in 2 cases. One patient had liver metastases that couldn’t be detected on PET scan alone and required combination with CT for anatomical registration.

From the 2 first studies, limited information could be gathered regarding the utility of PET/CT for posttreatment surveillance. One study demonstrated reliable PET/CT performance, with a total of 8 PET/CT performed, from which 6 patients were true negatives and 2 patients were true positives for nasal disease.^56^ However, the second study considered PET/CT to be unreliable for posttreatment surveillance, since both patients included required a biopsy to confirm or refute complete remission of disease.^54^

The last article interestingly compared the SUV_max_ values of PET/CT for SL and SCC.^58^ The study concluded that no significant differences could be found between the values for each type of neoplasm.

**Take-home points:** PET/CT yielded good results for initial staging of SL, with avid FDG uptake in the nasal cavity and paranasal sinuses. Therapy response assessment with PET/CT was also evaluated in a study, and it showed a promising ability to distinguish between persistent disease and low-grade inflammatory uptake. Evidence for the posttreatment surveillance ability of PET/CT is still limited.

### Miscellaneous Articles

A total of 3 studies were included discussing the utility of other radiotracers in the management of sinonasal malignancies (Table 8).^16,59,60^ The first study compared FDG-PET to ^11^C-choline PET scan and concluded that both radiotracers could help distinguish SCC from benign lesions through higher SUV values. However, when looking at all malignant tumors, FDG uptake was variable with some cases of adenocarcinoma with low uptake. In comparison, ^11^C-choline uptake was less variable for malignant tumors. Furthermore, for malignant lesions with low FDG uptake, ^11^C-choline uptake was consistently higher. Therefore, ^11^C-choline PET may be a useful complement to FDG-PET in cases of low uptake of FDG to identify malignant tumors. The second article assessed the role of ^11^C-methionine as a radiotracer to assess skull base tumors. For qualitative assessments of skull base tumors, ^11^C-methionine PET/CT showed superior interrater agreement and had higher uptake for tumors at the skull base than FDG-PET/CT, making it possibly a superior imaging modality. The third article evaluated the utility of ^68^Gallium-DOTATATE PET/CT in small cell neuroendocrine carcinoma (SNEC) and ENB. ^68^Gallium-DOTATATE is a radiotracer used to identify somatostatin receptor positive neuroendocrine tumors, making it potentially useful for cases of SNEC and ENB. Although the article only included 6 cases, the authors discussed how ^68^Gallium-DOTATATE PET/CT allowed for detection of an unknown primary, identified metastasis missed by conventional imaging and identified abnormal tracer uptake that was not present with FDG PET/CT. The authors thus concluded that ^68^Gallium-DOTATATE PET/CT could be useful for SNEC and ENB in detecting metastases, primary lesions, and recurrence.

**Table 8.** Summary of Included Studies Discussing PET Radiotracers for Sinonasal Malignancies.

Author (year)	Study objective	Imaging modality	Sample size	SUV_max_ (range)	Findings	Type of study (level of evidence)	Bias assessment
Ninomiya (2004)	Evaluate clinical utility of ^11^C-choline PET scan for tumors of the nasal cavity and paranasal sinuses	^11^C-choline PET FDG-PET	8 sinonasal malignancies 14 benign lesions	^11^C-choline PET 0.35-8.80 FDG-PET 0.53-19.36	^11^C-choline PET may a supporting tool for cases of low uptake on FDG-PET in the nasal cavity and paranasal sinuses	Retrospective 4	Serious
Tomura (2016)	Compare MET-PET/CT with FDG-PET/CT in tumors of the skull base	MET-PET/CT FDG-PET/CT	9 SCC 5 ACC 1 ENB 1 Chondrosarcoma 1 MMM 10 Other skull base tumors	n/a	MET-PET/CT has potential for imaging of skull base tumors	Retrospective 4	Serious
Liu (2021)	Assess the value of ^68^Gallium-DOTATATE PET/CT in sinonasal neuroendocrine tumor management	^68^Gallium-DOTATATE PET/CT FDG-PET/CT	6 sinonasal neuroendocrine tumors	n/a	^68^Gallium-DOTATATE PET/CT could be useful in detecting metastases, primary lesions, and recurrences	Retrospective 4	Serious

One final article was included discussing clinical implications of incidental sinonasal positive FDG uptake on PET/CT.^22^ It was concluded that incidental significant FDG uptake in the sinonasal cavities is at high risk (40%) of being neoplastic, but that a diagnostic biopsy is advocated in all cases for further workup (Table 9).

**Table 9.** Summary of Findings for Each Designated Section.

Section	Main findings	Types of studies (level of evidence)	Bias assessment
**Inverted papilloma and squamous cell carcinoma**	Both benign IPs and IPs with SCC present high SUV values. There is high variability in SUV values of benign IPs and overlap with SUV values of IPs harboring SCC. Statistical comparison made between IPs and sinonasal malignancies yielded insufficient results for clear differentiation on sole basis of SUV values. PET/CT may detect IP recurrence after surgical resection.	1 Prospective (3b) 6 Retrospective (4)	1 Moderate 6 Serious
**Sinonasal carcinoma**			
Detection and initial staging	FDG-PET/CT may be useful for initial detection of primary tumors, neck, and distant metastases. Reporting of diagnostic performance indices showed sensitivity for primary tumor assessment ranging from 77% to 100% across studies and specificity from 73% to 100%. Additional clinically relevant information after CT and MRI were performed was obtained only for assessment of distant metastases and second primary tumor. PET scan failed to reveal orbital or dural involvement and regional neck metastases. FDG-PET scan is unable to detect lesions close to the brain due to physiologic FDG uptake.	2 Prospective (3b) 6 Retrospective (4)	2 Moderate 6 Serious
Characterization of FDG avidity with SUV values	Information concerning the type of tumor may be obtained from SUV value. ENB, MPNST, and JNA may present with lower SUV values on average, with ENB SUV values ranging from 4.6 to 10.7. SNUC and SCC may present with higher SUV value on average, respectively, 7.6 to 52.9 and 2.4 to 26.1.	6 Retrospective (4)	6 Serious
Prognosis	Higher SUV value may correlate with poorer outcome for cancer recurrence and death, regardless of conventional staging. TLG may be a useful parameter to evaluate disease survival.	7 Retrospective (4)	7 Serious
Surveillance	PET/CT may be a valuable tool for posttreatment detection of locoregional recurrences and distant metastases. FDG-PET/CT is unable to detect brain metastases on follow-up.	1 Prospective (3b) 3 Retrospective (4)	1 Moderate 3 Serious
**Sinonasal melanoma**	PET scan was useful for detecting primary tumors. PET/CT may be the superior imaging modality over PET scan, CT, or MRI alone, for combined metabolic and anatomical information. PET scan or PET/CT may detect locoregional recurrences and distant metastases on follow-up. The role of PET scan in differentiating postsurgical inflammation from local recurrence was limited. It was also unable to detect cerebral metastases.	4 Retrospective (4)	4 Serious
**Sinonasal lymphoma**	PET/CT may be a valuable tool for initial staging of SL, showing avid FDG uptake in the nasal cavity and paranasal sinuses, ranging from 5 to 25. PET/CT may also be useful for therapy response assessment, having the ability to distinguish persistent disease from low-grade inflammatory uptake. There is limited evidence for the posttreatment surveillance ability of PET/CT.	3 Retrospective (4)	3 Serious
**Miscellaneous**	Three different radiotracers were tested. ^11^C-Choline had less SUV variability than FDG and may be useful for suspected malignant lesions with low FDG uptake. Methionine may be a superior radiotracer for skull base tumors. ^68^Gallium-DOTATATE-PET/CT may be a superior imaging modality for SNEC and ENB, as it was superior in detecting metastases, primary lesions, and recurrences in these types of cancers.	4 Retrospective (4)	4 Serious

**Take-home points:** ^11^C-choline had less SUV variability than FDG and showed promising results for malignancies with low FDG uptake. ^11^C-Methionine had higher uptake for malignancies at the skull base region. ^68^Gallium-DOTATATE was superior to FDG for neuroendocrine tumors such as SNEC and ENB, both in detection of distant metastases and primary lesions and recurrences.

## Discussion

To our knowledge, this is the first systematic review investigating the role of PET scan in the management of sinonasal malignancies. Although PET scans are commonly used in head and neck oncology, due to the rarity of sinonasal malignancies, there appears to be a lack of consensus on the role of the imaging modality for this subset of tumors. The objective of this study was to review the literature systematically and provide a summary for clinicians who wish to better understand how to integrate the imaging modality in the management of sinonasal malignancies and know the limitations of the imaging modality for these types of tumors.

Through the qualitative assessment of the included articles, 5 recurrent themes and potential roles of PET scans were uncovered for the management of sinonasal malignancies. These include tumor detection and initial staging, characterization of metabolic activity, prognosis, treatment planning, and re-staging or surveillance. Although, most studies had samples with different types of sinonasal tumors, a subset of studies had samples which only included cases of IP and SCC, melanoma, and lymphoma. Results of the systematic review were thus presented using this structure.

The present review highlighted the usefulness of PET for sinonasal malignancies in a variety of domains while being less helpful for other applications. First, regarding distinguishing benign IP from IP with SCC, most authors agree that the use of PET scan should be done cautiously, as there is an overlap in SUV values between both disease entities [13-16]. For the initial detection of sinonasal malignancies, PET scans have a good sensitivity, ranging from 77% to 100%, while its specificity was slightly lower, from 73% to 100%.^23–27^ Similar results were found for regional and distant metastasis.^27^ However, PET scans do not appear to add clinically relevant information to the primary tumor assessment with conventional imaging, such as dural or orbital involvement, especially because of normal metabolic brain activity obfuscating the visibility of these tumors close or extending into the brain.^23–26,29^ Rather, PET scans appeared to contribute clinically additional information for distant metastasis and finding second primary tumors.^28,29^ With respect to characterizing FDG avidity with SUV values, it was shown that some types of tumors could possibly have a higher or lower FDG uptake on average. ENB, MPNST, and JNA were all found to have a lower FDG uptake on PET scan, ENB being between 4.6 and 10.7, while SNUC and SCC had an SUV ranging respectively from 7.6 to 52.9 and 2.4 to 26.1.^16,22,31,32^ For prognosis, based on retrospective studies, certain parameters such as SUV value and TLG at initial staging could be linked with some outcomes such as DSS and RFS.^35,36^ Although PET scans have been shown to have the potential to change the accuracy and field of radiation for head and neck cancers, no studies had cohorts composed exclusively of patients with sinonasal malignancies. For surveillance, PET was valuable in the posttreatment detection of locoregional recurrences and detection of distant metastases, but the timing of posttreatment PET scan was still uncertain across the studies.^23,24,45,46^ In addition, no cerebral metastases could be accurately detected on follow-up using PET scan, and MRI was required for detection.^23,24^

PET scans also appeared valuable in the management of sinonasal melanoma with good diagnostic performance indices for detection of the primary tumor, which could depend on the size, shape, and location of the lesion.^50,51^ Similarly for surveillance, PET scans appeared to be valuable for distant metastases detection, in addition to detection of locoregional recurrences.^50^ In the case of lymphoma, PET scans demonstrate value for initial staging and for treatment response prediction, but not for surveillance.^54,56,58^

Across the included studies, there are important limitations to be addressed in future research. First, there is a lack of standardized SUV cutoff value suggesting whether a sinonasal lesion is malignant.^36,39^ Many studies also showed the inability of PET scans to distinguish postsurgical inflammation from disease recurrence,^50,56^ highlighting the necessity to find a good posttreatment timing for PET to avoid unclear findings. For distant metastases, FDG-PET scans also had trouble detecting brain metastases,^23,24,50,51^ due to the high background activity in this region and diffuse bone marrow involvement.^56^ Importantly, primary lesions close to the brain also couldn’t be detected by FDG-PET, once again showing that normal metabolic activity of the brain interferes with visualization of primary tumor.^23–26,29^

This study also has its own set of limitations. To begin with, most articles that met inclusion criteria were retrospective studies with lower levels of evidence and serious risk of bias. Some articles also had a total of 3 or 4 patients and therefore provided limited evidence. Furthermore, given that most studies had different objectives with varying outcomes of interest, results could not be aggregated together and summarized quantitatively. Lastly, this study only highlights the key findings of the different articles that were included. All details and nuances from individual studies could not be included in this review.

## Conclusions

The present study assesses the utility of PET scans in the management of sinonasal malignancies and analyzes its role in different areas of cancer care. The relevance of this study is rooted in the present lack of consensus regarding when to use PET scans in cases where sinonasal malignancy is present or suspected. PET scan is a powerful diagnostic tool of significant value for most head and neck malignancies, and its role for sinonasal malignancies has yet to be clarified. Further research can help to clarify the value of PET scans for these patients.
